# Skeletal Muscle Transcriptomic Comparison Between Men and Women in Response to Acute Sprint Exercise

**DOI:** 10.3389/fgene.2022.860815

**Published:** 2022-07-12

**Authors:** Mingkun Nie, Qingling Liu, Cheng Yan

**Affiliations:** ^1^ School of Physical Education, Xinxiang University, Xinxiang, China; ^2^ School of Pharmacy, Xinxiang University, Xinxiang, China; ^3^ Key Laboratory of Nano-carbon Modified Film Technology of Henan Province, Xinxiang University, Xinxiang, China; ^4^ Diagnostic Laboratory of Animal Diseases, Xinxiang University, Xinxiang, China

**Keywords:** muscle, KEGG, acute sprint exercise, hub genes, sex-biased genes

## Abstract

**Background:** Acute sprint exercise is a time-efficient physical activity that improves cardiorespiratory fitness in younger and middle-aged adults. Growing evidence has demonstrated that acute sprint exercise provides equal to or superior health benefits compared with moderate-intensity continuous training, which will dramatically increase aerobic capacity, insulin sensitivity, and muscle capillarization. Although the beneficial effects of acute sprint exercise are well documented, the mechanisms behind how acute sprint exercise prevents disease and benefits health are less understood.

**Method:** We obtained differentially expressed genes in muscle (vastus lateralis) from men and women before and after an acute sprint exercise. Then, we identified hub genes from the protein–protein interaction (PPI) network of differentially expressed genes (DEGs) and key transcription factors in men and women related to acute sprint exercise. Finally, Kyoto Encyclopedia of Genes and Genomes (KEGG) and Gene Ontology (GO) enrichment analyses are performed on DEGs and sex-biased genes, respectively.

**Results:** First, we identified 127 sexually dimorphic genes in men (90 upregulated and 37 downregulated) and 75 genes in women (90 upregulated and 37 downregulated) in response to acute sprint exercise. Second, CEBPB, SMAD3, and CDKN1A are identified as the top three hub genes related to men-biased genes. Accordingly, the top three hub genes related to women-biased genes are JUN, ACTB, and SMAD7. In addition, CLOCK, ZNF217, and KDM2B are the top three enriched transcriptional factors in men-biased genes, while XLR, SOX2, JUND, and KLF4 are transcription factors enriched most in women-biased genes. Furthermore, based on GO and KEGG enrichment analyses, we identified potential key pathways in regulating the exercise-related response in men and women, respectively.

**Conclusion:** In this study, we found the difference in gene expression and enrichment pathways in muscle in men and women in response to acute sprint exercise. These results will shed new light on the mechanism underlying sex-based differences in skeletal muscle remodeling and metabolism related to acute sprint exercise, which may illustrate the mechanisms behind how acute sprint exercise prevents disease and benefits health.

## Introduction

Regular physical activity is well accepted as a key intervention for reducing the risk of chronic disease, including mobility disability, heart disease, type 2 diabetes, and several cancers (Hawley, 2004; Warburton et al., 2006). Acute sprint exercise is a time-efficient physical activity that improves cardiorespiratory fitness in younger and middle-aged adults (Willoughby et al., 2016), promotes favorable enhancement in insulin sensitivity (Richards et al., 2010), and has beneficial effects on the enhancement of cognitive abilities with increased neuroprotective factors (Kujach et al., 2019). The previous study has shown that acute sprint exercise provides equal to or superior health benefits compared with moderate-intensity continuous training, which will dramatically increase aerobic capacity, insulin sensitivity, and muscle capillarization (Cocks et al., 2016). Different from other exercise manners, acute sprint exercise is characterized by rapid glycogen degradation and large net ATP breakdown in muscle and is accompanied by the increased secretion of hormones such as insulin, catecholamines, and growth factor (Esbjörnsson-Liljedahl et al., 1985; Esbjörnsson et al., 2009). Gene signatures related to a turnover of skeletal muscle mass are upregulated after sprint exercise (Rundqvist et al., 2019). Although the beneficial effects of acute sprint exercise are well documented, the mechanisms behind how acute sprint exercise prevents disease and benefits health are less understood.

Skeletal muscles have the fourth largest number of sex differentially expressed genes across 29 human tissues, with more than 3,000 differentially expressed genes between male and female skeletal muscles at rest (Welle et al., 2008; Lopes-Ramos et al., 2020). Thus, differentially expressed genes may be the most important cause of sex differences in muscle morphology, function, and plasticity (Pointer et al., 2013). According to a previous study, men usually have significantly larger cross-sectional areas than women, especially in type II fibers (Miller et al., 1993). Conversely, women may be more resistant to muscle fatigue due to a higher proportion of type I fibers (Salomoni et al., 2008; Avin et al., 2010). Furthermore, men and women have sex-based differences in response to sprint interval training. It is reported that after six weeks of sprint interval training, insulin sensitivity was increased only in healthy men but not women (Metcalfe et al., 2012). Sprint interval training reduced 24-h blood glucose concentration in men but not women among adults with overweight (Gillen et al., 2014). Glucose transporter 4 (GLUT4), a key regulator of glucose metabolism in muscle, is higher increased in men compared with women in response to sprint interval training. In summary, previous studies indicate that men showed stronger adaptative responses to sprint interval training than women. However, the mechanism underlying sex-based differences in skeletal muscle remodeling and metabolism in response to acute sprint exercise is still unclear. To the best of our knowledge, the difference of global gene expression in skeletal muscle between men and women in response to once acute sprint exercise (three bouts of 30-s sprint exercise with 20-min rest) has not been previously demonstrated.

In this study, we focused on the response to acute sprint exercise, not long-term adaptation exercise training, to increase the understanding of the transcriptomic difference of skeletal muscle in response to acute sprint exercise between men and women. First, we obtained differentially expressed genes in muscle from men and women before and after an acute sprint exercise. Then, we identified hub genes from the protein–protein interaction (PPI) network of DEGs in men and women related to acute sprint exercise. Finally, KEGG and GO enrichment analyses were performed on DEGs and sex-biased genes, respectively. Our study shed light on the understanding of how the health benefits of acute sprint exercise are regulated in men and women. In addition, our results may also indicate the mechanism underlying sex-based differences in skeletal muscle remodeling and metabolism related to acute sprint exercise.

## Materials and Methods

### Acquisition of Expression Profile

The gene expression profiles related to acute sprint exercise are downloaded from GSE126296 (PMID: 31647849), which contains the expression data of quadriceps femoris muscle (vastus lateralis) from seven males and seven females before and after acute sprint exercise (Rundqvist et al., 2019). As described in the original study, participants have to be in good general health, participate in leisure-time sports but not at an elite level, and be between 20 and 30 years of age. The participants with chronic illness, acute infection, pregnancy, severe asthma, or use of nicotine-containing products are excluded.

### Identification of Differentially Expressed Genes

Differentially expressed genes are identified with “limma” package in R software. The screening threshold used in this study is set at FDR <0.05 and |log_2_ (fold change)| > 0.3. Heat maps and volcano maps were visualized by employing the “ggplot2” and “pheatmap” packages in R software.

### Construction of Protein–Protein Interaction Network and Identification of Hub Genes

A PPI network is constructed based on the STRING database. To identify hub genes, we use the radiality algorithm in cytoHubba plug-in in Cytoscape.

### Kyoto Encyclopedia of Genes and Genomes, Gene Ontology, and Transcription Factor Enrichment Analyses

KEGG, GO, and transcription factor analyses are performed using the Enrichr tool (https://maayanlab.cloud/Enrichr/).

## Results

### Analysis of Differentially Expressed Genes in Men and Women in Response to Acute Sprint Exercise

**FIGURE 1:**
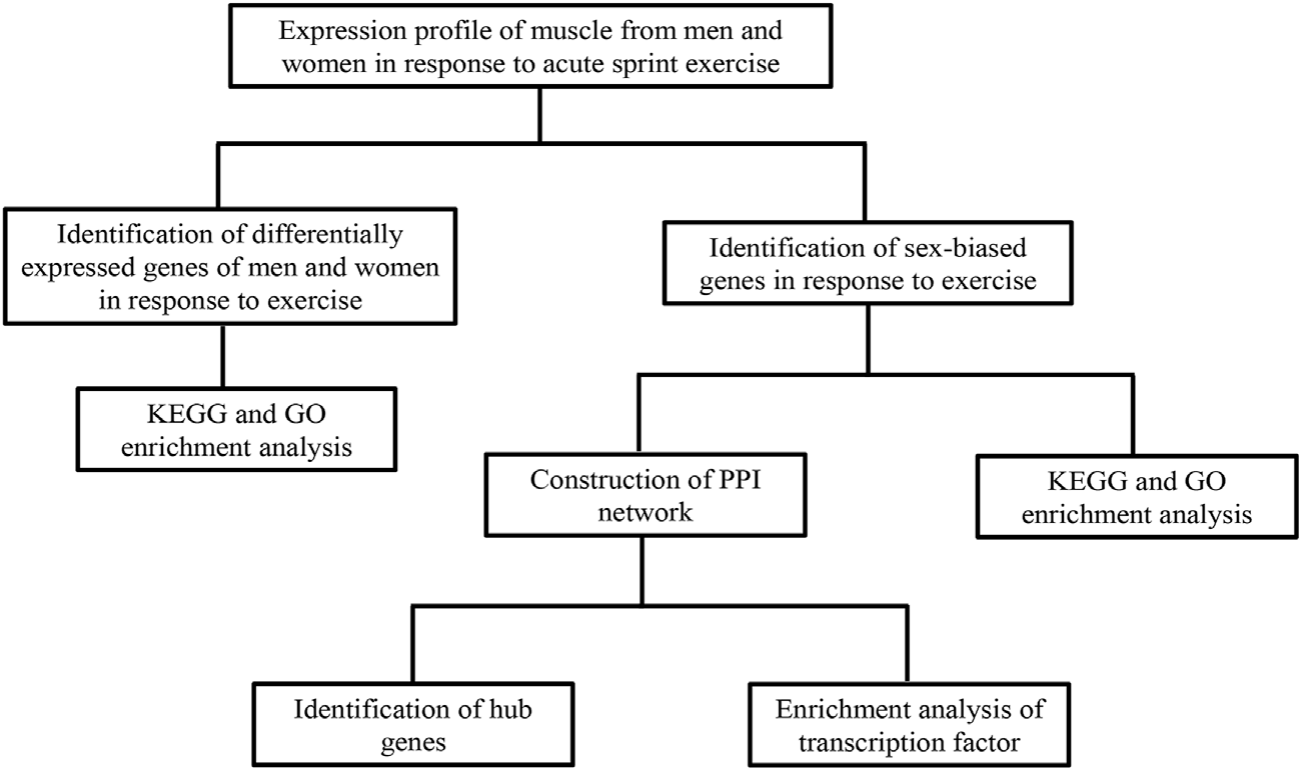
A flowchart of analytical approaches used in this study. GEO, Gene Expression Omnibus; GO, Gene Ontology; KEGG, Kyoto Encyclopedia of Genes and Genomes.

**FIGURE 2:**
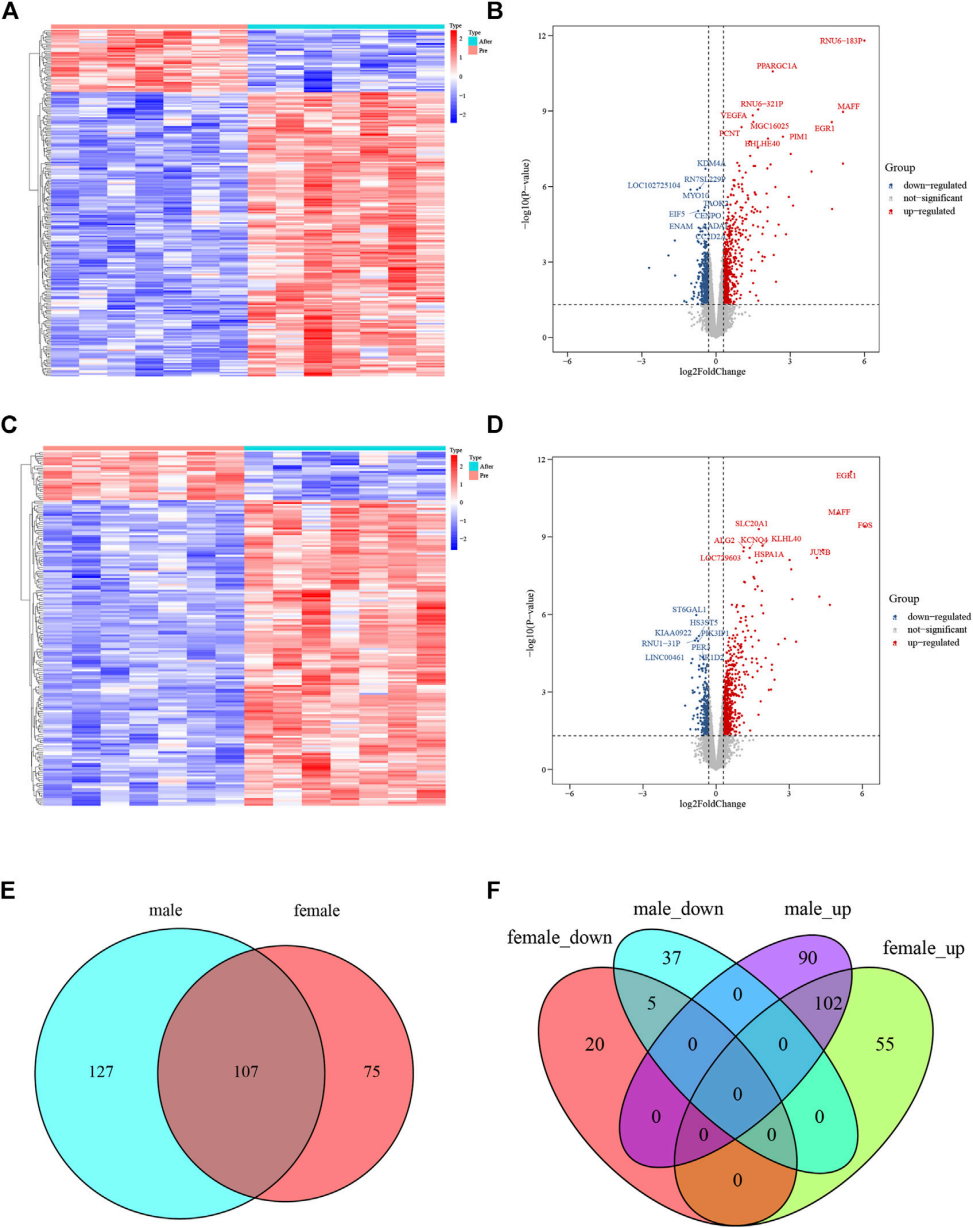
Analysis of differentially expressed genes in response to acute sprint exercise in skeletal muscle from men and women. **(A)** Heat map of 234 differentially expressed genes in muscle from men. **(B)** Volcano plot of 234 differentially expressed genes in muscle from men. **(C)** Heat map of 182 differentially expressed genes in muscle from women. **(D)** Volcano plot of 182 differentially expressed genes in muscle from women. **(E)** Venn diagram showing the overlap of differentially expressed genes between men and women. **(F)** Venn diagrams showing the overlap of the differentially expressed genes that were upregulated and downregulated in men and women. Red dots represent upregulated genes, and blue dots indicate downregulated genes. FC, fold change.

A flowchart of analytical approaches used in this study is provided in Figure 1. In order to identify the differentially expressed genes in skeletal muscle between men and women in response to acute sprint exercise, we performed bioinformatics analysis on leg skeletal muscle collected from human subjects before and after three all-out cycle sprints. We set the threshold at FDR <0.05 and | log_2_ (fold change) | > 0.3. A total of 234 genes (192 upregulated and 42 downregulated) in men were identified as differentially expressed genes in response to acute sprint exercise (Supplementary Table S1). The heat map and volcano plot for differentially expressed genes are shown in Figures 2A and B. Accordingly, 182 differentially expressed genes are identified in women, containing 157 upregulated and 25 downregulated genes (Supplementary Table S2). The heat map and volcano plot for differentially expressed genes are shown in Figures 2C and D. Furthermore, we identified 127 sexually dimorphic genes in men (90 upregulated and 37 downregulated) and 75 genes in women (55 upregulated and 20 downregulated) in response to acute sprint exercise (Figures 2E,F) (Supplementary Tables S3, S4). These results demonstrate that numerous sexually dimorphic genes are differentially expressed in men and women, and the amount of men-biased genes is much greater than that of women-biased genes in response to acute sprint exercise.

### Identifying Hub Genes and Transcription Factors Associated With Sex-Specific Expression Signatures in Response to Acute Sprint Exercise

**FIGURE 3:**
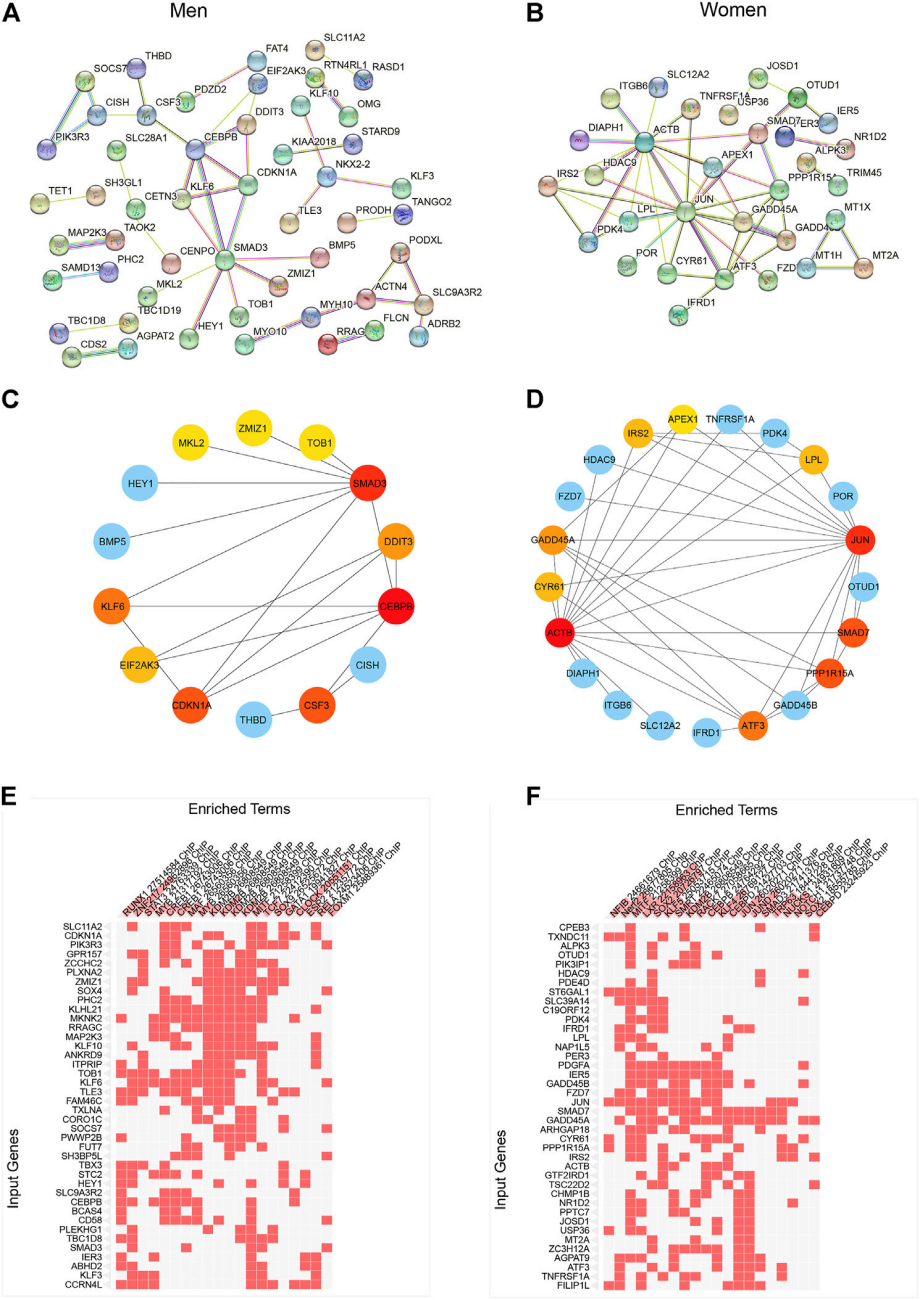
Construction of PPI network and identification of differentially expressed genes in men and women. **(A)** PPI network of differentially expressed genes in men. **(B)** PPI network of differentially expressed genes in women. **(C)** Hub gene network of differentially expressed genes in men. **(D)** Hub gene network of differentially expressed genes in women. **(E)** Transcription factor enrichment of men-biased genes. **(F)** Transcription factor enrichment of women-biased genes.

In order to explore the network of sexually dimorphic genes in response to acute sprint exercise, we constructed the PPI networks based on male-biased genes and female-biased genes, respectively (Figures 3A,B). Hub genes from the PPI network were screened out with cytoHubba application in Cytoscape using the radiality method (Figures 3C,D). The top three hub genes related to men-biased genes are CEBPB, SMAD3, and CDKN1A. Accordingly, the top three hub genes related to women-biased genes are JUN, ACTB, and SMAD7. These genes may encode core proteins with important biological regulatory functions resulting in sex-based differences in skeletal muscle remodeling and metabolism in response to acute sprint exercise. Subsequentially, we performed transcription factor enrichment analysis to provide insights into the underlying change of transcription activity in sex-specific expression signatures. CLOCK, ZNF217, and KDM2B are the top three enriched transcriptional factors in men-biased genes (Figure 3E; Supplementary Table S5), while XLR, SOX2, JUND, and KLF4 are transcription factors enriched most in women-biased genes (Figure 3F; Supplementary Table S6). These results suggest that sexually dimorphic genes may be regulated by different transcription factors in response to acute sprint exercise.

### Gene Ontology and Kyoto Encyclopedia of Genes and Genomes Enrichment Analyses of Differentially Expressed Genes in Men and Women in Response to Acute Sprint Exercise

**FIGURE 4:**
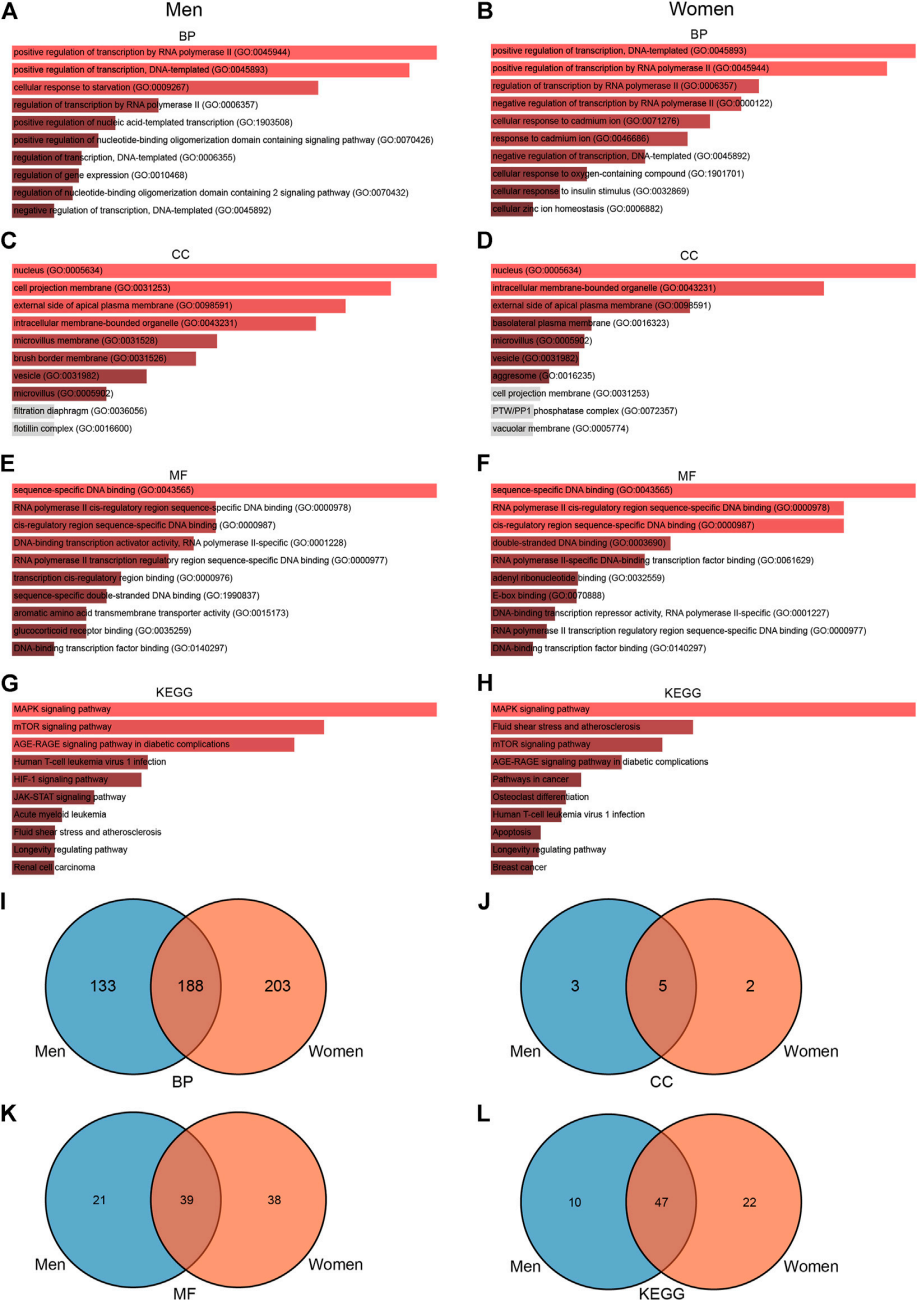
GO and KEGG enrichment analyses of DEGs in men and women. **(A)** BP enrichment analyses of differentially expressed genes in men. **(B)** BP enrichment analyses of differentially expressed genes in women. **(C)** CC enrichment analyses of differentially expressed genes in men. **(D)** CC enrichment analyses of differentially expressed genes in women. **(E)** MF enrichment analyses of differentially expressed genes in men. **(F)** MF enrichment analyses of differentially expressed genes in women. **(G)** KEGG enrichment analyses of differentially expressed genes in men. **(H)** KEGG enrichment analyses of differentially expressed genes in women. **(I)** Venn diagram showing the overlap of BP pathways between men and women. **(J)** Venn diagram showing the overlap of CC pathways between men and women. **(K)** Venn diagram showing the overlap of MF pathways between men and women. **(L)** Venn diagram showing the overlap of KEGG pathways between men and women. GO, Gene Ontology; BP, biological process; CC, cellular component; MF, molecular function; KEGG, Kyoto Encyclopedia of Genes and Genomes; TF, transcriptional factor.

To further explore the functions associated with DEGs, we conducted a series of enrichment analyses. GO enrichment analysis showed that 389 terms were significantly enriched (*p*-value < 0.05) in men, including eight cellular component terms, 60 molecular function terms, and 321 biological process terms (Figures 4A,C,E). Notable pathways among BP were positive regulation of transcription by RNA polymerase II, positive regulation of transcription, DNA-templated, and cellular response to starvation. In the molecular function category, the top three terms were sequence-specific DNA binding, RNA polymerase II *cis*-regulatory region sequence-specific DNA binding, and *cis*-regulatory region sequence-specific DNA binding. Moreover, nucleus, cell projection membrane, and external side of apical plasma membrane were the top three significant terms in the cellular component category. KEGG pathway analysis identified 57 significantly enriched pathways such as MAPK signaling pathway, mTOR signaling pathway, and AGE-RAGE signaling pathway in diabetic complications (Figure 4G), while GO enrichment analysis in women showed that 176 terms were significantly enriched (*p*-value < 0.05), including seven cellular component terms, 77 molecular function terms, and 392 biological process terms (Figures 4B,D,F). The BPs of the DEGs from the female subjects were primarily enriched in positive regulation of transcription, DNA-templated, positive regulation of transcription by RNA polymerase II, and regulation of transcription by RNA polymerase II. The CC results revealed that DEGs in the female cohorts were mainly enriched in nucleus, intracellular membrane-bounded organelle, and external side of apical plasma membrane. The MF results indicated that DEGs identified in the female cohorts were primarily enriched in sequence-specific DNA binding, RNA polymerase II *cis*-regulatory region sequence-specific DNA binding, and *cis*-regulatory region sequence-specific DNA binding. KEGG pathway analysis identified 69 significantly enriched pathways in female, including MAPK signaling pathway, fluid shear stress, and mTOR signaling pathway (Figure 4H). Furthermore, we use the Venn diagram to indicate the overlap of items of GO and KEGG between men and women (Figures 4I–L, Supplementary Tables S5, S6). These results indicate that changes of signaling pathways are different in skeletal muscle between men and women in response to acute sprint exercise.

### Gene Ontology and Kyoto Encyclopedia of Genes and Genomes Enrichment Analyses of Male-Biased and Female-Biased Genes in Response to Acute Sprint Exercise

**FIGURE 5:**
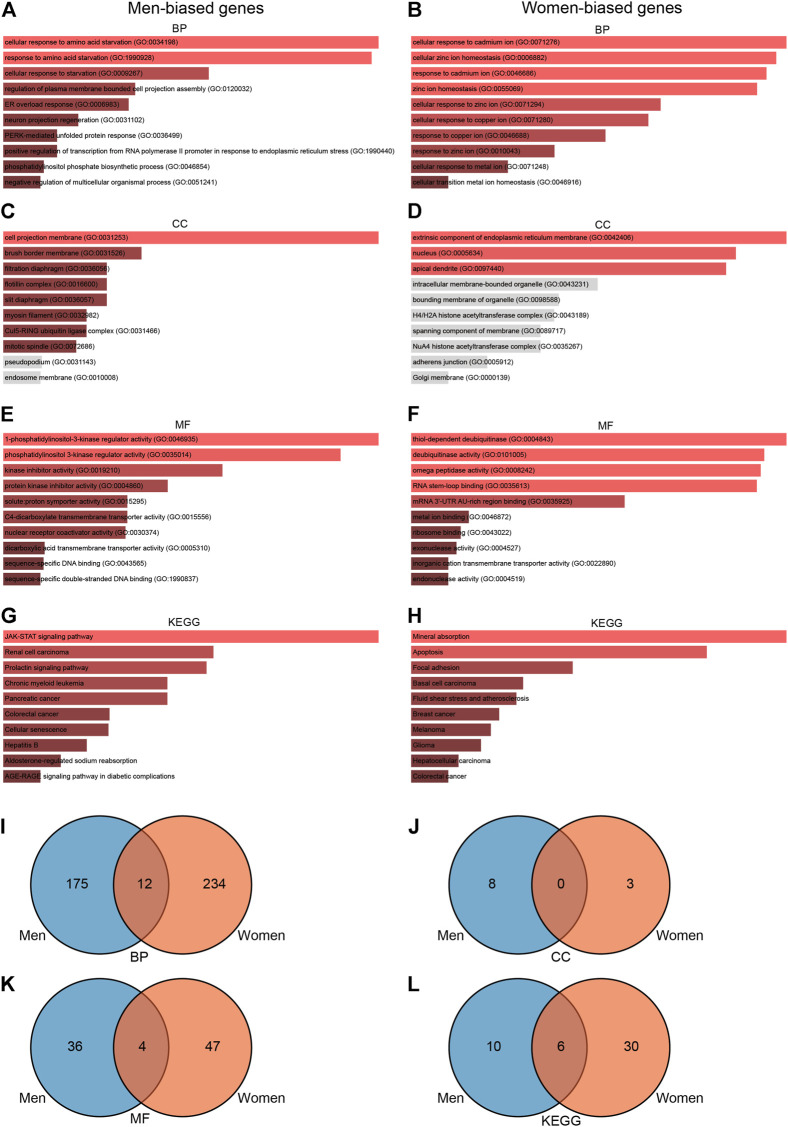
GO and KEGG enrichment analyses of sexually dimorphic genes in men and women. **(A)** BP analyses of sexually dimorphic genes in men. **(B)** GO pathway analyses of sexually dimorphic genes in women. **(C)** CC pathway analyses of sexually dimorphic genes in men. **(D)** CC pathway analyses of sexually dimorphic genes in women. **(E)** MF enrichment analyses of sexually dimorphic genes in men. **(F)** MF enrichment analyses of sexually dimorphic genes in women. **(G)** KEGG enrichment analyses of sexually dimorphic genes in men. **(H)** KEGG enrichment analyses of sexually dimorphic genes in women. **(I)** Venn diagram showing the overlap of BP pathways between men-biased and women-biased genes. **(J)** Venn diagram showing the overlap of CC pathways between men-biased and women-biased genes. **(K)** Venn diagram showing the overlap of MF pathways between men-biased and women-biased genes. **(L)** Venn diagram showing the overlap of KEGG pathways between men-biased and women-biased genes. GO, Gene Ontology; BP, biological process; CC, cellular component; MF, molecular function; KEGG, Kyoto Encyclopedia of Genes and Genomes; TF, transcriptional factor.

To further explore the functions associated with sex-biased genes, we conducted a series of enrichment analyses. GO enrichment analysis showed that 235 terms were significantly enriched (*p*-value < 0.05) in men, including eight cellular component terms, 40 molecular function terms, and 187 biological process terms (Figures 5A,C,E). Notable pathways among BP were cellular response to amino acid starvation, response to amino acid starvation, and cellular response to starvation. In the molecular function category, the top three terms were 1-phosphatidylinositol-3-kinase regulator activity, phosphatidylinositol 3-kinase regulator activity, and kinase inhibitor activity. Moreover, cell projection membrane, brush border membrane, and filtration diaphragm were the top three significant terms in the cellular component category. KEGG pathway analysis identified 16 significantly enriched pathways such as JAK-STAT signaling pathway, renal cell carcinoma, and prolactin signaling pathway (Figure 5G). GO enrichment analysis in women showed that 300 terms were significantly enriched (*p*-value < 0.05), including three cellular component terms, 51 molecular function terms, and 246 biological process terms (Figures 5B,D,F). The BPs of the DEGs from the female subjects were primarily enriched cellular response to cadmium ion, cellular zinc ion homeostasis, and response to cadmium ion. The CC results revealed that DEGs in the female cohorts were mainly enriched in extrinsic component of endoplasmic reticulum membrane, nucleus, and apical dendrite. The MF results indicated that DEGs identified in the female cohorts were primarily enriched in thiol-dependent deubiquitinase, deubiquitinase activity, and omega peptidase activity. KEGG pathway analysis identified 36 significantly enriched pathways in females, including mineral absorption, apoptosis, and focal adhesion (Figure 5H). Furthermore, we used the Venn diagram to indicate the overlap of items of GO and KEGG between men- and women-biased genes (Figures 5I–L; Supplementary Tables S7, S8). These results indicate that the signaling pathways enriched by men-biased and women-biased genes are different.

## Discussion

Acute sprint exercise induces numerous adaptations in the body resulting in beneficial effects on health, which may induce fat loss, increase cognitive functions, improve cardiorespiratory fitness, and alleviate metabolic syndrome (Gibala et al., 2006; Vollaard and Metcalfe, 2017; Kujach et al., 2019). Although the effects of sprint exercise-induced adaptations to skeletal muscle have been well-established in various diseases, the difference of global gene expression in skeletal muscle in men and women in response to acute bouts of acute sprint exercise has not been previously demonstrated. In this study, we conducted an analysis of gene expression profiles obtained from muscle biopsies collected from human volunteers before and after acute bouts of acute sprint exercise to discover exercise-related sexually dimorphic genes, which might be part of the mechanism underlying the sex-based differences in skeletal muscle remodeling and metabolism in response to acute sprint exercise.

RNA-seq results show that about 3,000 genes of human in skeletal muscle at rest are identified as differentially expressed genes (Lindholm et al., 2014). However, the sex-specific epigenomic signatures of immediate early genes in response to acute sprint exercise are still unknown. We performed differentially expressed gene analysis to explore the difference in gene expression between men and women in response to acute sprint exercise. A total of 234 genes (192 upregulated and 42 downregulated) in men were identified as differentially expressed genes using bioinformatics analysis, while only 182 differentially expressed genes were identified in women, containing 157 upregulated and 25 downregulated genes. The number of differentially expressed genes in men is dramatically greater than that in women. This result may account for the fact that men have a stronger adaptative response to sprint interval training than women. Cytokine-inducible SH2-containing protein (CISH), known as cytokine-induced STAT inhibitor, is the top upregulated gene in males. A recent study showed that CISH may play an important role in promoting free fatty acid mobilization (Vestergaard et al., 2014; Vendelbo et al., 2015). Our data also suggest that the expression of CISH may be also regulated by sex hormones. After acute sprint exercise, OTUD1 is the top fold-change gene in females. Although previous independent studies showed that OTUD1 was significantly induced by acute sprint exercise, its function related to acute sprint exercise was still unknown (Bolotta et al., 2020). Furthermore, we identified ZNF217 and CLOCK as the top significant enrichment transcription factors in men-biased genes. CLOCK is necessary for the maintenance of the skeletal muscle phenotype and function through regulating MyoD. Skeletal muscles from mice with mutation of Clock and Bmal1 exhibit ∼30% reductions in normalized maximal force (Andrews et al., 2010). Our result is consistent with previous research that CLOCK is a key regulator of muscle fibers, which may be one of the reasons inducing the difference in muscle type between men and women. However, LXR and SOX2 are the top significant enrichment transcription factors in women. LXRβ is the dominant LXR subtype in skeletal muscle regulating lipogenesis and cholesterol efflux (Hessvik et al., 2010). This result indicates that transcription factors induced by acute sprint are different between men and women. Since the biological function of ZNF217 and SOX2 in skeletal muscle is still unclear, we can only speculate that interference of these two factors may play a role in muscle groups of different fiber type composition or metabolism in men or women, respectively.

Our study also showed that men and women shared several GO and KEGG pathways in response to acute sprint exercise. The significantly enriched GO terms for DEGs in men and women can be categorized into several biological functional groups including positive regulation of transcription, cellular response to starvation, and positive regulation of nucleic acid-templated transcription. These results indicate that acute sprint exercise promoted cell proliferation in muscle both in men and women, which is consistent with the previous study (Pearson, 1990; Bazgir et al., 2017; Saito et al., 2020). Moreover, DEGs in men and women significantly enrich a list of KEGG pathways relevant to the MAPK signaling pathway, mTOR signaling pathway, and AGE-RAGE signaling pathway. The AKT/mTOR signaling pathway played an important role in regulating muscle hypertrophy (Bodine et al., 2001). Similar to AKT/mTOR signaling, MAPK also promoted improvements in fuel homeostasis and could prevent skeletal muscle atrophy (Kramer and Goodyear, 1985). What is more, emerging evidence shows that RAGE signaling pathway is critical for skeletal muscle physiology controlling both the activity of muscle precursors during skeletal muscle development and the correct time of muscle regeneration after acute injury (Riuzzi et al., 2018). These data may implicate a mechanism behind muscle growth both in men and women.

Exercise training is associated with profound changes in skeletal muscle, including increased abundance of glucose up-take, activation of AMPK, and mitochondrial biogenesis (Egan and Zierath, 2013). Our results also provide novel insights into the difference in the pathway between men and women in response to acute sprint. Compared with women-biased genes, the enrichment pathways of men-biased genes are mainly about response to starvation or amino acid starvation (GO: cellular response to amino acid starvation). Men always consume more energy than women. Higher enrichment of these pathways may support an increased rate of energy consumption in men. Therefore, men are more urgent to face the “insufficient energy” situation. However, there is a tendency for women to restore the calcium and zinc homeostasis (GO: cellular response to cadmium ion). These results indicate acute sprint may induce a more severe disturbance of ion homeostasis in cellular processes. Moreover, our study also identified novel sex-specific areas, such as cellular response to cadmium ion, that exist in the fine regulatory system of the muscle response to acute sprint exercise at the transcriptional level which merit further exploration. It is well known that gender differences exist in skeletal muscle mass and distribution (Janssen et al., 1985). Females often have less total and lean body mass, a higher body fat percentage, and a smaller muscle fiber cross-sectional area (Staron et al., 1985). KEGG analysis showed that the JAK-STAT signaling pathway is dramatically enriched in the men-biased genes, not in women. The JAK/STAT pathway participates in lipid metabolism in muscle. Activation of the JAK/STAT pathway in skeletal muscle promotes muscle hypertrophy by increasing the proliferation of satellite cells (Moresi et al., 2019). These results indicate that the JAK-STAT pathway may account for gender differences in muscle fiber-type distribution and substrate metabolism in men and women. These findings may also help us to understand how sex differences manifest in gene regulatory networks in muscle.

Sample size significantly affects DEG analysis output in microarray datasets, and more biological samples predict more accurate outcomes (Stretch et al., 2013). The gene expression profile we analyzed is from previously published literature. In the original study, Rundqvist et al. (2019) expressed reservations about the identification of sexually dimorphic changes due to the small sample size. As described in the method, we downloaded the expression profile of muscle from the original dataset including seven males and seven females before and after acute sprint exercise. Then, we identified the differentially expressed genes using the “limma” package. The screening threshold used in this study was FDR<0.05 and |log2 (fold change)| > 0.3. Through this manner, we identified a total of 234 DEGs in men and 182 DEGs in women. We believe that the biological number in our study (7 vs. 7) is sufficient to get the significant DEGs to a certain extent. We also believe that more samples included will increase the statistical power and further improve the conclusion in the future.

This study needs to be improved in the future for the following reasons: first, the protein level of differentially expressed genes remains to be confirmed. Second, further studies are required to understand the role of sex-biased genes in response to acute sprint exercise. Third, even if the health benefits brought by sprint exercise are similar to those of endurance training, it also should be mentioned that acute response to exercise is not always similar to the long-term adaptation exercise training.

## Conclusion

In this study, we found sex-biased genes and their related potential key pathways in muscle in response to acute sprint exercise. Thus, these results will shed new light on the mechanism underlying sex-based differences in skeletal muscle remodeling and metabolism related to sprint interval training, which may also illustrate the mechanisms behind how acute sprint exercise prevents disease and benefits health.

## Data Availability

The datasets presented in this study can be found in online repositories. The names of the repository/repositories and accession number(s) can be found in the article/Supplementary Material.

## Abbreviations

AGE, advanced glycation end; BP, biological process; CC, cellular component; CISH, cytokine-inducible SH2-containing protein; DEGs, differentially expressed genes; FDR, false discovery rate; GO, Gene Ontology; KEGG, Kyoto Encyclopedia of Genes and Genomes; MF, molecular function; PPI, protein–protein interaction; RAGE, receptor for advanced glycation end.
